# Enhanced Degradation of Oxytetracycline Antibiotic Under Visible Light over Bi_2_WO_6_ Coupled with Carbon Quantum Dots Derived from Waste Biomass

**DOI:** 10.3390/molecules29235725

**Published:** 2024-12-04

**Authors:** Haitao Ren, Fan Qi, Ke Zhao, Du Lv, Hao Ma, Cheng Ma, Mohsen Padervand

**Affiliations:** 1 Technological Institute of Materials & Energy Science (TIMES), Xijing University, Xi’an 710123, China; 2 School of Environmental Science and Engineering, Shaanxi University of Science and Technology, Xi’an 710021, China; 3 State Key Laboratory of Medicinal Chemical Biology, College of Pharmacy, Nankai University, Tianjin 300071, China; 4 Department of Chemistry, Faculty of Science, University of Maragheh, Maragheh P.O. Box 55181-83111, Iran

**Keywords:** visible-light photocatalysis, antibiotic contamination, carbon quantum dots, waste biomass, degradation mechanism

## Abstract

Improving the photogenerated carrier separation efficiency of individual semiconductor materials has always been a key challenge in photocatalysis. In this study, we synthesized a novel photocatalytic material, N-CQDs/UBWO, in situ by combining nitrogen-doped carbon quantum dots (N-CQDs) derived from discarded corn stover with ultrathin Bi_2_WO_6_ nanosheets (UBWO). Detailed characterization indicates that the random distribution of N-CQDs on the UBWO surface increases the specific surface area of UBWO, which is beneficial for the adsorption and degradation of oxytetracycline (OTC). More importantly, N-CQDs act as electron acceptors, promoting the effective separation of photogenerated charges, prolonging the lifetime of charge carriers in UBWO, and thereby enhancing the degradation efficiency of OTC. As a result, the optimized 3wt%N-CQDs/UBWO could degrade 85% of OTC within 40 min under visible light, with a removal rate four times that of pure Bi_2_WO_6_. The performance of photocatalytic degradation over OTC by 3wt%N-CQDs/UBWO exceeds that of most reported Bi_2_WO_6_-based photocatalysts. The EPR analysis confirmed that ∙O_2_^−^ and ∙OH are the main active species in the photocatalytic degradation of OTC on 3wt%N-CQDs/UBWO. This study provides insight into designing green, low-cost, and efficient photocatalysts using CQDs derived from waste biomass and the degradation of emerging pollutants like antibiotics.

## 1. Introduction

Antibiotics, as the commonly used drugs for treating human diseases, inevitably generate a large amount of threatful wastewater during their production and practical uses [1,2]. In recent years, the overuse of antibiotics has exacerbated this phenomenon and related concerns. Antibiotics, as one of the four predominant pollutants, are discharged into the environment without effective purification, posing serious menaces to the ecological environment and human life and health [3,4]. Therefore, there is an urgent need to develop economically efficient technologies aimed at treating antibiotic wastewater. Visible light-driven photocatalytic technology is regarded as a promising solution for pollutant elimination, attracting widespread attention in recent years due to its advantages, including low energy consumption, mild reaction conditions, and environmentally friendly cleaning [5,6]. However, the low reaction efficiency of most photocatalytic materials greatly limits the application of photocatalytic technology in antibiotic pollution remediation [7]. Therefore, researchers are more committed to developing low-cost, durable, and highly efficient photocatalytic constructions.

Among various photocatalysts, Bi_2_WO_6_, as a representative bismuth-based semiconductor with a unique layered structure, shows high stability, low conduction band position, and suitable bandgap (2.6–2.8 eV), leading to great potential in the field of photocatalytic environmental purification [8,9]. However, due to the improper visible light harvesting performance (λ ≤ 450 nm) and high electron–hole recombination rate, the intrinsic Bi_2_WO_6_ photocatalytic material shows limited applications in removing antibiotics [10,11]. To date, researchers have made many efforts, including constructing heterojunctions, introducing defects, controlling morphology, and loading co-catalysts to improve the photocatalytic performance of Bi_2_WO_6_ [12,13]. Although these strategies can effectively improve the photocatalytic performance of Bi_2_WO_6_, they still face problems such as poor material tolerance and high preparation cost, which hinder the development of Bi_2_WO_6_-based heterojunction photocatalysts [14,15]. The main challenge of Bi_2_WO_6_-based photocatalytic composites is how to improve the separation of photogenerated charge carriers, reduce the preparation/utilization costs, improve tolerance, and achieve its large-scale application in environmental purification.

CQDs, as a new type of carbon nanomaterials, are composed of sp2/sp3 hybridized carbon cores and surface functional groups [16,17]. Due to their low toxicity, low cost, and good conductivity, they have been widely used in designing efficient photocatalytic constructions, such as CQDs/Bi_2_WO_6_, CQDs/TiO_2_, CQDs/Bi_2_MoO_6_, CQDs/BiOIO_3_, CQDs/ZnIn_2_S_4_, and CQDs/g-C_3_N_4_ [6,18,19,20]. CQDs are used as co-catalysts in these systems to expand the pho-to-responsive range and facilitate photogenerated charge migration [21,22]. Therefore, the use of CQDs instead of metal oxide semiconductors or precious metals to improve the photocatalytic performance of semiconductor materials has attracted huge attention in recent years [23]. China generates approximately 900 million tons of waste biomass annually. Suppose these biomasses can be converted into CQDs for the design of novel efficient photocatalysts. In that case, it can not only minimize the use of toxic and/or expensive chemicals but also promote the green and low-cost preparation of photocatalytic materials.

In this work, N-CQDs, derived from discarded corn stalks, were added to the Bi_2_WO_6_ hydrothermal synthesis system for the in situ formation of the novel ultrathin CQDs/Bi_2_WO_6_ composite material. The microstructure of the photocatalysts was systematically explored using a multi-technique approach. The results showed that N-CQDs were tightly and randomly dispersed on the surface of UBWO nanosheets, leading to the significant improvement of charge separation efficiency in UBWO. The optimized 3wt%N-CQDs/UBWO composite system can effectively degrade 85% of OTC within 40 min under visible light, with a first-order rate constant 4.0 times higher than that of Bi_2_WO_6_. This work provides a meaningful reference for the design of green and low-cost Bi_2_WO_6_-based composite photocatalysts with eminent purification capability of antibiotic wastewater.

## 2. Results and Discussion

### 2.1. Structural Characterization of the Photocatalysts

**Figure 1:**
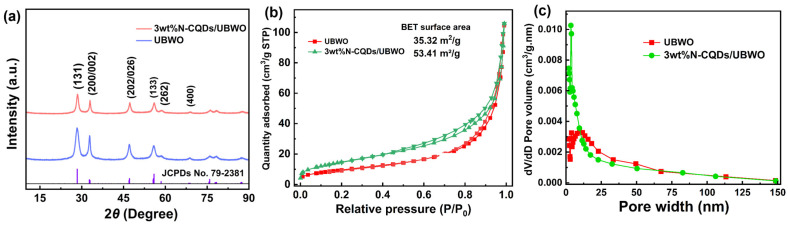
(**a**) XRD patterns; (**b**) N_2_ adsorption–desorption; (**c**) pore diameter distribution of 3wt%N-CQDs/UBWO and UBWO.

The XRD analysis was performed at room temperature to identify the crystalline phases of the structures. As shown in Figure 1a, all diffraction peaks belong to Bi_2_WO_6_, well in agreement with the standard JCPDS: 79-2381 [24], indicating that the insertion of N-CQDs does not affect the crystal structure of UBWO. The intensity of the 3wt%N-CQD/UBWO diffraction peak is significantly lower than that of UBWO at the same 2θ position, which may be ascribed to the relatively low crystallinity of N-CQDs [25]. The efficiency of photocatalysts is closely related to their corresponding specific surface area. Therefore, the porosity of UBWO and 3wt%N-CQDs/UBWO were specifically explored by BET-BJH analysis techniques. Figure 1b shows that the N_2_ adsorption–desorption isotherm exhibits a typical IV hysteresis loop, implying the micro-porosity and meso-porosity of the designed photocatalyst [26]. The specific surface areas of UBWO and 3wt%N-CQDs/UBWO are 35.32 and 53.41 m^2^/g, respectively, indicating that the introduction of N-CQDs endows UBWO with a larger specific surface area, which is beneficial for exposing a larger number of photocatalytic active sites in the reaction. The corresponding pore size distribution map is shown in Figure 1c, which indicates that the pore size of the two catalysts is almost less than 50 nm, predominantly consisting of microporous and mesoporous channels. The 3wt%N-CQDs/UBWO exhibits a larger pore volume (0.164 cm^3^/g), providing sufficient active sites for pollutant adsorption and degradation reactions.

**Figure 2:**
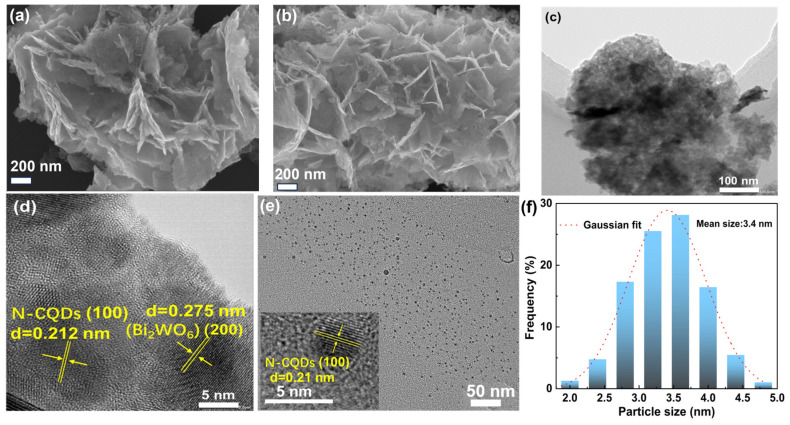
SEM images of (**a**) UBWO and (**b**) 3wt%N-CQDs/UBWO. (**c**) TEM and (**d**) HR-TEM images of 3wt%N-CQDs/UBWO. (**e**) TEM image of N-CQDs prepared from discarded corn stover (inset is the HRTEM image of an individual N-CQDs). (**f**) Particle size distribution diagram of N-CQDs.

The SEM image of UBWO is shown in Figure 2a, demonstrating its irregular porous structure composed of nanosheets. The microstructure of 3wt%N-CQDs/UBWO composite material was also investigated by SEM. As shown in Figure 2b, the SEM images reveal that the photocatalytic composite possesses an irregular structure randomly composed of ultrathin nanosheets, similar to blooming petals, with abundant porous regions between the nanosheets. The above results indicate that N-CQDs regulate the morphology of UBWO during hydrothermal processes, resulting in a larger specific surface area and more surface-active sites. Due to the small size of N-CQDs, they were not observed in the SEM images. To demonstrate the successful construction of a composite system from N-CQDs and UBWO, TEM images of the optimized 3wt%N-CQDs/UBWO were also provided. As shown in Figure 2c, the TEM image shows a large number of N-CQDs small particles on the surface of 3wt%N-CQDs/UBWO. From the HR-TEM image of the optimized composite photocatalyst (Figure 2d), the 3wt%N-CQDs/UBWO surface exhibits two different lattice fringes. The first one has a fringe spacing of 0.21 nm, corresponding to the lattice spacing of the N-CQDs (100) crystal plane, and the other region has a stripe spacing of 0.275 nm, attributing to the lattice spacing of the Bi_2_WO_6_ (200) crystal plane [27]. This result indicates that N-CQDs have been successfully loaded onto the surface of Bi_2_WO_6_ nanosheets. TEM and size distribution of N-CQDs derived from discarded corn stover were investigated to describe the morphology. As displayed in Figure 2e, the TEM image shows that the shape of N-CQDs is spherical, with completely dispersed particles without any aggregation. The HR-TEM image of N-CQDs in the inset of Figure 2e exhibits the distinct lattice fringes with a lattice spacing of 0.212 nm, corresponding to the lattice spacing of the graphite carbon (100) plane [28]. The particle distribution histogram of N-CQDs is shown in Figure 2f, which shows that the particle size of N-CQDs ranges from 1.8 to 4.9 nm, with an average size of 3.4 nm. The above results indicate that the 3wt%N-CQDs/UBWO composite photocatalytic system was successfully constructed using N-CQDs derived from discarded corn stover.

**Figure 3:**
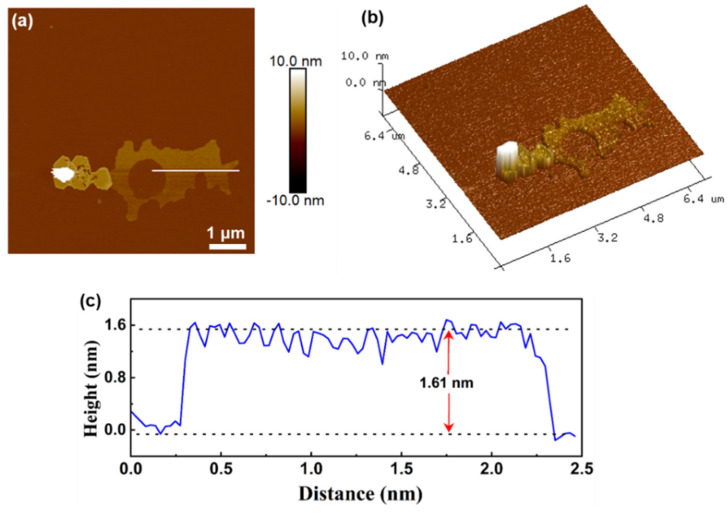
AFM (**a**) 2D image; (**b**) 3D image; (**c**) corresponding thickness profiles of ultrathin UBWO.

AFM analysis was conducted to measure the surface topology of the ultrathin Bi_2_WO_6_ (UBWO) nanosheet structures. As shown in Figure 3a,b, UBWO has an irregular two-dimensional nanosheet structure, and the thickness measurements were performed at a randomly selected location on its surface. Figure 3c shows that the average thickness of UBWO nanosheets is approximately 1.6 nm, fully demonstrating the synthesis of UBWO under CTAB-mediated hydrothermal conditions. Compared with bulk or particulate Bi_2_WO_6_, ultrathin UBWO can shorten the distance of charge transfer during the photocatalytic process, reduce the recombination rate of electrons and holes, and improve its photocatalytic performance [29].

**Figure 4:**
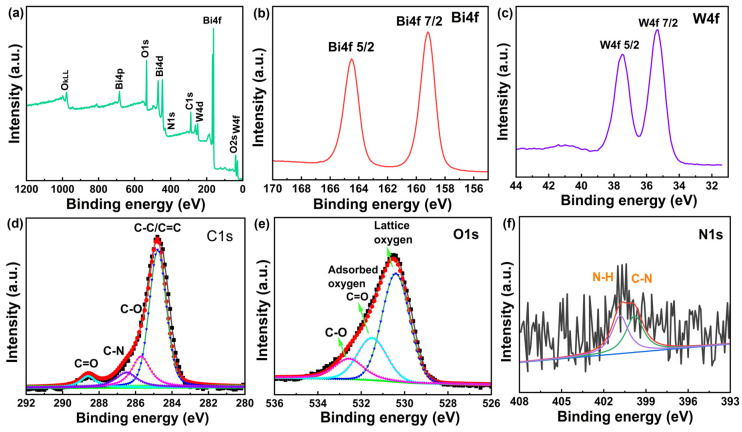
(**a**) XPS survey spectrum; (**b**) Bi4f spectrum; (**c**) W4f spectrum; (**d**) C1s spectrum; (**e**) O1s spectrum; (**f**) N1s spectrum of 3wt%N-CQDs/UBWO.

XPS technology was used to interpret the chemical state and surface elemental composition of 3wt%N-CQDs/UBWO. Figure 4a shows the XPS full spectrum of 3wt%N-CQDs/UBWO, which is composed of Bi, W, C, O, and N elements. In Figure 4b, the two peaks at 159.2 eV (Bi4f 7/2) and 164.5 eV (Bi4f 5/2) in the Bi4f spectrum were attributed to typical Bi^3+^ in Bi_2_WO_6_. In Figure 4c, the XPS spectrum of W4f can be deconvoluted into two peaks at 37.5 and 35.3 eV for W4f 5/2 and W4f 7/2, respectively. The XPS spectrum of C1s in Figure 4d shows four peaks at 284.8, 285.6, 286.7, and 288.0 eV, respectively, corresponding to the C-C/C=C, C-O, C-N, and C=O bonds in N-CQDs [30]. The XPS spectrum of O1s exhibits three peaks at 530.4, 531.4, and 532.7 eV, respectively, corresponding to lattice oxygen (Figure 4e), adsorbed oxygen or C=O, and C-O bond [31]. As shown in Figure 4f, the XPS spectrum of N1s in 3wt%N-CQDs/UBWO exhibits two peaks at 399.8 and 400.6 eV, respectively, attributed to the C-N and N-H bonds in N-CQDs. The above results indicate that N-CQDs and UBWO have been successfully coupled through chemical interactions, which is beneficial for achieving the separation and migration of photogenerated carriers.

### 2.2. Degradation of Oxytetracycline

**Figure 5:**
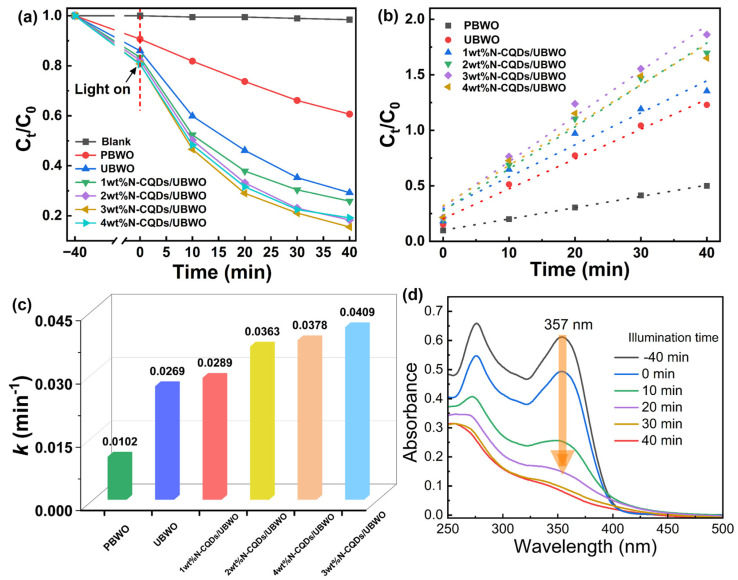
(**a**) Performance curves and (**b**,**c**) first-order rate constants of different photocatalysts for degradation of OTC. (**d**) UV-Vis absorption spectra of OTC solution at different photocatalytic reaction times.

The photocatalytic efficiency of the synthesized catalyst was assessed for the tetracycline antibiotics OTC degradation as a typical pollutant under visible light. As shown in Figure 5a, in the absence of a photocatalyst, the concentration of OTC solution remains almost unchanged after long-term irradiation, which means that the self-decomposition ability of OTC can be ignored. The degradation rate of OTC by PBWO was 40% within 40 min, while the degradation rate of OTC by UBWO reached 70% within the same reaction time. Indeed, the ultrathin structure of UBWO can shorten the distance of charge migration in the photocatalytic process and reduce the probability of electron–hole recombination [32].

After introducing the optimal amount of N-CQDs on the surface of UBWO, the OTC degradation rate was 85% after 40 min. It can be seen that the degradation efficiency over the composite photocatalysts increases first and then decreases with the increase in CQD loading. Introducing an appropriate amount of CQDs may be beneficial for improving the light-harvesting properties and separation efficiency of photogenerated charges in UBWO. However, the excessive CQDs can block the active sites of UBWO and hinder its light absorption, thereby reducing the photocatalytic activity towards OTC removal [33].

According to the quasi-first-order kinetic model, −ln(C_t_/C_0_) = *k*t, the first-order rate constant (*k*) for OTC degradation was determined (Figure 5b,c). The rate constant *k* = 0.0409 min^−1^ for the optimal catalyst, 3wt%N-CQDs/UBWO, is 4.0 times that of PBWO (*k* = 0.01628 min^−1^). The enhanced photocatalytic performance of 3wt%N-CQD/UBWO could be attributed to the presence of N-CQD as an efficient electron acceptor in the structure, which promotes the rapid separation of photogenerated carriers in UBWO. Figure 5d shows the UV-Vis absorption spectra of treated OTC solution by 3wt%N-CQDs/UBWO under visible light. It can be detected that with the extension of illumination time, the UV-Vis absorption peak of OTC solution at 357 nm gradually decreases, indicating that 3wt% N-CQDs/UBWO can effectively degrade the OTC molecules under visible light. Furthermore, Table 1 demonstrates a comparison of the performance of the prepared 3wt%N-CQDs/UBWO photocatalyst in degrading antibiotics with the current literature reports. Obviously, 3wt%N-CQDs/UBWO exhibits a better degradation performance for tetracycline antibiotics under visible light, manifesting that the developed 3wt%N-CQDs/UBWO photocatalyst has great practical application prospects.

### 2.3. Degradation Mechanism of Oxytetracycline

**Figure 6:**
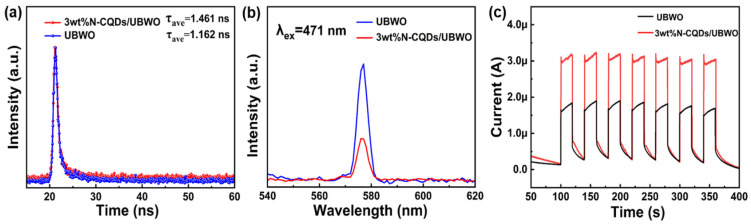
(**a**) The time-resolved PL spectra; (**b**) PL spectra; (**c**) photocurrent responses of UBWO and 3wt%N-CQDs/UBWO.

The kinetics of photogenerated carrier transfer and separation in UBWO and 3wt%N-CQDs/UBWO were studied by time-resolved PL spectroscopy, steady-state PL, and photocurrent response. After fitting the attenuation curve of Figure 6a with the parameters in Table 2, the fluorescence lifetimes of UBWO and 3wt%N-CQDs/UBWO were measured to be 1.162 and 1.461 ns, respectively, manifesting that the composite system shows a good carrier separation behavior and prolongs the lifetime of photogenerated charges [44]. The PL spectrum (Figure 6b) shows that 3wt%N-CQDs/UBWO possesses a lower PL intensity than UBWO, implying its higher electron–hole separation efficiency compared to the latter case [45]. The photocurrent response curve (Figure 6c) shows that the transient photocurrent of 3wt%N-CQDs/UBWO is significantly higher than that of UBWO, indicating that the composite system has excellent photoelectric conversion characteristics that arise from the higher separation efficiency of photogenerated charges in the photocatalytic composite [46]. The above results reveal that a favorable heterostructure is formed between N-CQDs and UBWO, which promotes the separation and migration of photogenerated carriers.

**Figure 7:**
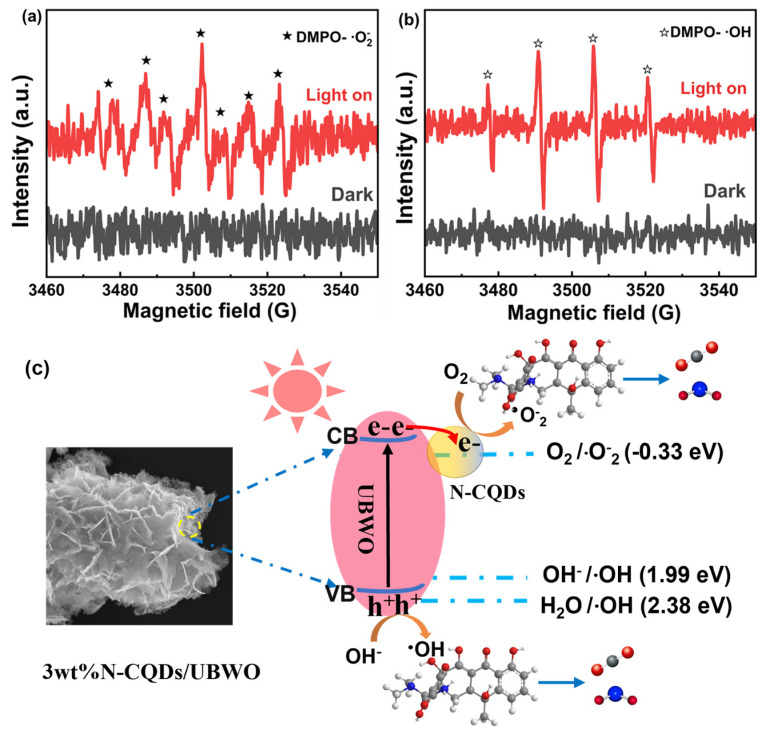
EPR signals of 3wt%N-CQDs/UBWO: (**a**) DMPO—∙O_2_^−^ and (**b**) DMPO—∙OH under visible light. (**c**) Schematic diagram of the degradation mechanism of antibiotics by 3wt%N-CQDs/UBWO under visible light.

The active species in the photocatalytic degradation of OTC by 3wt%N-CQDs/UBWO were identified using EPR, and 5,5-dimethyl-1-pyrroline-N-oxide (DMPO) was employed as the spin-trapping agent, where ∙O_2_^−^ and ∙OH were collected in methanol and H_2_O, respectively. As shown in Figure 7a,b, no significant free radical signal was detected in 3wt%N-CQDs/UBWO under dark conditions. However, four strong peaks of DMPO- ∙OH with an intensity ratio of 1:2:2:1 and six signal peaks of DMPO- ∙O_2_^−^ were detected in 3wt%N-CQDs/UBWO under visible light, proving that it can simultaneously generate ∙O_2_^−^ and ∙OH radicals under visible light. The production of these reactive oxygen species is beneficial for the rapid photodegradation of OTC. Moreover, the possible charge transfer mechanism between N-CQDs and UBWO was analyzed thermodynamically based on the conditions under which active species are generated. As shown in Figure 7c, the excited electron (e^−^) transfers from the valence band (VB) of UBWO to its conduction band (CB) under visible light irradiation. Due to the π-conjugated structure of the carbon nucleus inside N-CQDs, they could be excellent electron acceptors [47]. Therefore, when N-CQDs are in close contact with UBWO, the e^−^ on the CB of UBWO can easily transfer to N-CQDs, thereby achieving effective separation of photogenerated charges in UBWO. The band structure of UBWO was determined based on the results of the analogous reference literature [48]. Due to the more negative CB position of UBWO compared to O_2_/∙O_2_^−^ (−0.33 V vs. NHE), the photogenerated e^−^ on N-CQDs can reduce the surface adsorbed O_2_ to ∙O_2_^−^ [49]. In addition, the hole (h^+^) on VB of UBWO can oxidize the hydroxyl ions to ∙OH. The h^+^ can also directly react with the OTC molecules [50]. Finally, the 3wt%N-CQDs/UBWO heterojunction produced abundant ∙O_2_^−^, ∙OH, and h^+^ active species under visible light, which together reacted with the OTC molecules to decompose them into the organic small molecules, CO_2_ and H_2_O.

## 3. Materials and Methods

### 3.1. Materials and Chemicals

Urea, oxytetracycline, and 5,5-dimethyl-1-pyrroline-N-oxide (DMPO) were purchased from Aladdin Industrial Corp. (Shanghai, China). Sodium tungstate dihydrate, bismuth nitrate pentahydrate, and hexadecyl trimethyl ammonium bromide (CTAB) were supplied by Macklin Reagent Co., Ltd., (Shanghai, China). The chemical and reagents were all of analytical grade and did not require any additional purification.

### 3.2. Preparation of Samples

Synthesis of N-CQDs derived from discarded corn stover: Waste corn stover collected in farmland was washed twice with ultrapure water and dried at 80 °C for 6 h. The dried corn stover was ground into powder. A total of 0.3 g of straw powder and 0.1 g of urea were mixed in 60 mL of ultrapure water. After stirring for 1 h, the mixture was loaded into a 100 mL hydrothermal reactor and treated at 200 °C for 10 h. After completion of the reaction, the liquid was filtered three times through a 50 nm filter membrane to obtain a transparent yellow-brown solution. It was dried at 80 °C for 12 h to obtain N-CQDs powder.

**Scheme 1:**
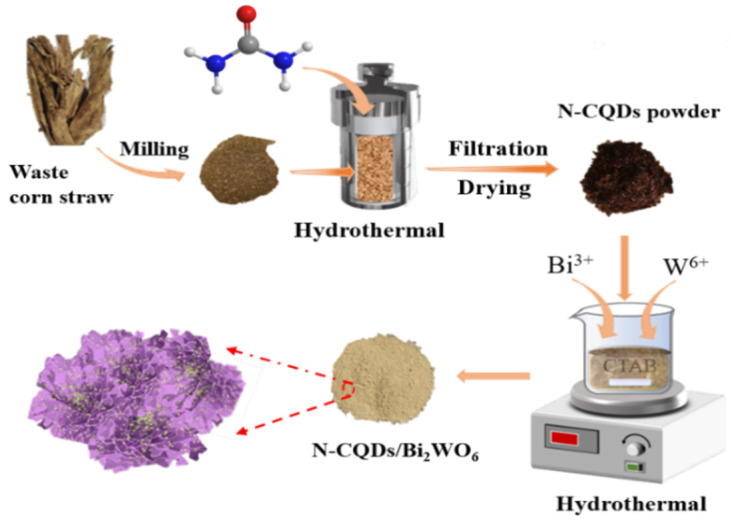
Schematic description of the synthesis of N-CQDs and N-CQDs/Bi_2_WO_6_.

Preparation of N-CQDs/BiW_2_O_6_ photocatalytic composite: A total amount of 0.33 g of Na_2_WO_4_·2H_2_O, 0.97 g of Bi(NO_3_)_3_·5H_2_O, and 50 mg of CTAB were added to 75 mL of ultrapure water. After 1 h of magnetic stirring, 7, 14, 21, and 28 mg of the as-synthesized N-CQDs were added to the medium. The reaction mixture was further stirred for another hour, transferred into a 100 mL high-pressure vessel lined with polytetrafluoroethylene, and heated at 120 °C for 24 h. After the reaction was completed, the product was washed 5 times with ultrapure water and anhydrous ethanol and dried at 80 °C for 10 h to obtain the final sample. The synthesis process is schematically shown in Scheme 1, where the samples obtained by adding 7, 14, 21, and 28 mg of N-CQDs are named 1wt%N-CQDs/UBWO, 2wt%N-CQDs/UBWO, 3wt%N-CQDs/UBWO, and 4wt%N-CQDs/UBWO, respectively. The preparation of the bulk Bi_2_WO_6_ (PBWO) sample is similar to the above steps, except that CTAB was not added.

### 3.3. Characterization and Instruments

High-resolution transmission electron microscopy (HR-TEM) was implemented on an FEI Talos F200i field emission electron microscope at 200 kV. Scanning electron microscopy (SEM) was performed on a scanning electron microscopy (Hitachi S-4800, Tokyo, Japan). The atomic force microscope (AFM) measurement was performed on a MultiMode 8 Bruker’s microscope with the Nanoscope V controller. The specific surface area was analyzed using Brunauer–Emmett–Teller (BET, ASAP 2460) with N_2_ as an adsorbate gas. Powder X-ray diffraction (XRD) patterns were performed on a D/Max-2400 X-ray diffractometer with Cu *K*α radiation (Japan Rigaku, Tokyo, Japan). The photoluminescence (PL) spectra were carried out using an Edinburgh Instruments FS5 spectrofluorometer (Edinburgh, UK); lifetime was obtained on the same FS5 Edinburgh Instruments equipped with an EPLED-340 pulsed source. The electron paramagnetic resonance (EPR) spectra were obtained from a Bruker A300 instrument (BRUKER, Mannheim, Germany).

The transient photocurrent responses were measured on an electrochemical system (CHI 660E, Shanghai Chenhua Instrument Co., Ltd., Shanghai, China). A typical three-electrode was immersed in a 0.5 mol/L Na_2_SO_4_ electrolyte solution, and a 300 W Xenon lamp was used as the light source. Fluorine-doped tin oxide (FTO) conductive glass (1 × 1 cm) was employed as the working electrode, and platinum sheet/calomel electrodes were used as the counter and reference electrodes, respectively.

### 3.4. Photodegradation Evaluation

An amount of 50 mL of OTC solution (20 mg/L) was mixed with 10 mg of catalysts under continuous stirring and allowed to reach adsorption equilibrium at dark after 40 min. For OTC degradation under light irradiation, the light source was a 300 W Xe lamp equipped with a Cut 420 nm filter to provide visible light. When the adsorption equilibrium was completed, the light source was turned on for OTC photodegradation. An amount of 3 mL of the reaction mixture was taken at different regular intervals and filtered using a 0.22 μm syringe to obtain the supernatant for determining the residual antibiotics concentrations. The measurements were performed on a UV-2600 UV–visible spectrophotometer (Shimadzu, Kyoto, Japan), with a maximum absorption wavelength of 357 nm.

The concentration of OTC was determined using the following formula:

$$\;\eta (\%)=(1\;\text{-}\frac{C}{C_{0}})\;\times \;100\%=(1\;\text{-}\frac{A}{A_{0}})\;\times \;100\%\;$$

where *C*_0_ represents the initial concentration, and *C* denotes the concentration at any time (*t*); *A*_0_ and *A* refer to the initial absorbance and absorbance at any time (*t*), respectively.

## 4. Conclusions

In summary, a novel N-CQDs/UBWO photocatalytic composite was constructed by the in situ combination of N-CQDs, derived from discarded corn stover, and UBWO via hydrothermal method. The optimized 3wt%N-CQDs/UBWO structure degraded 85% of OTC within 40 min under visible light, with a degradation rate four times that of pure Bi_2_WO_6_. Mechanism analysis and characterization showed that N-CQDs, as electron acceptors, promoted the effective separation of photoproduced charges in UBWO, thereby boosting the degradation efficiency of OTC by UBWO. In addition, the favorable dispersibility of N-CQDs hinders the aggregation of UBWO nanosheets, increases the specific surface area, and promotes the adsorption and degradation of OTC. The ultrathin structure of Bi_2_WO_6_ shortens the charge transfer distance and reduces the recombination probability of photoproduced carriers. Therefore, the synergistic effect of ultrathin structure and N-CQDs ameliorates the degradation performance of Bi_2_WO_6_ towards the OTC pollutant under visible light. This work provides a valuable reference for the rational design of green and low-cost photocatalysts alongside their excellent degradation capability of emerging pollutants such as antibiotics.

## Figures and Tables

**Table 1:** Comparison of tetracycline antibiotics degradation performance over 3wt%N-CQDs/UBWO and previously reported photocatalysts in literature.

Catalysts	Light Source	Photocatalytic Activity	Pollutant Concentration (mg/L)	Catalyst Dosage (g/L)	Ref.
BC-QDs@Bi_2_WO_6_	(λ > 420 nm)	80.0% (120 min)	20	0.5	[6]
B-TiO_2_	(λ > 400 nm)	66.2% (240 min)	10	0.2	[34]
CTOC/BaTiO_3_/CuS	(λ > 420 nm)	69.6% (60 min)	10	0.4	[35]
Bi_2_O_3_ QDs/g-C_3_N_4_	(λ > 400 nm)	82.5% (120 min)	10	0.5	[36]
2D/3D CN/CC-3	(λ > 420 nm)	69.6% (180 min)	10	0.5	[37]
UOCN-2	(λ > 410 nm)	87.2% (120 min)	30	0.4	[38]
2%CSs-Bi_2_WO_6_	(λ > 420 nm)	84.6% (60 min)	50	0.5	[39]
BiOBr/CQDs/g-C_3_N_4_	(λ > 420 nm)	82.7% (120 min)	20	0.2	[40]
ZnIn_2_S_4_/Bi_4_Ti_3_O_12_	(λ > 420 nm)	82.1% (60 min)	20	0.2	[41]
TF-Cu_2_O	(λ > 420 nm)	82.5% (60 min)	40	0.4	[42]
3wt%CQDs/Bi_2_WO_6_	(λ > 420 nm)	89.0% (40 min)	20	0.6	[43]
3wt%N-CQDs/UBWO	(λ > 420 nm)	85.0% (40 min)	20	0.2	This work

**Table 2:** Lifetime decay kinetic parameters of UBWO and 3wt%N-CQDs/UBWO.

Samples	Compounds	Lifetime (ns)	Relative Percentages (%)	Average Life (ns)	χ^2^
UBWO	τ_1_	0.15	64.98	1.162	1.137
	τ_2_	3.03	35.02		
3wt%N-CQDs/UBWO	τ_1_	0.2	67.76	1.461	1.096
	τ_2_	4.12	32.24		

τ: Decay lifetimes.

## Data Availability

Data are contained within the article.

## References

[1] Song Y., Zhang Z., Liu Y., Peng F., Feng Y. (2024). Enhancement of anaerobic treatment of antibiotic pharmaceutical wastewater through the development of iron-based and carbon-based materials: A critical review. J. Hazard. Mater..

[2] Chen Y., Jiang C., Wang Y., Song R., Tan Y., Yang Y., Zhang Z. (2022). Sources, environmental fate, and ecological risks of antibi otics in sediments of asia’s longest river: A whole-basin investigation. Environ. Sci. Technol..

[3] Zhang Z., Zhang Q., Wang T., Xu N., Luo T., Hong W., Penuelaso J., Gillingso M., Wang M., Gao W. (2022). Assessment of global health risk of antibioticresistance genes. Nat. Commun..

[4] Shao S., Hu Y., Cheng J., Chen Y. (2018). Degradation of oxytetracycline (OTC) and nitrogen conversion characteristics using a novel strain. Chem. Eng. J..

[5] Chen Y., Yang J., Zeng L., Zhu M. (2022). Recent progress on the removal of antibiotic pollutants using photocatalytic oxidation process. Crit. Rev. Environ. Sci. Technol..

[6] Tang X., Yu Y., Ma C., Zhou G., Liu X., Song M., Lu Z., Liu L. (2019). The fabrication of a biomass carbon quantum dot-Bi_2_WO_6_ hybrid photocatalyst with high performance for antibiotic degradation. New J. Chem..

[7] Zhou S., Jiang L., Wang H., Yang J., Yuan X., Wang H., Liang J., Li X., Li H., Bu Y. (2023). Oxygen Vacancies Modified TiO_2_/O-Terminated Ti_3_C_2_ Composites: Unravelling the Dual Effects between Oxygen Vacancy and High-Work-Function Titanium Carbide. Adv. Funct. Mater..

[8] Meng X., Zhang Z. (2016). Bismuth-based photocatalytic semiconductors: Introduction, challenges and possible approaches. J. Mol. Catal. A-Chem..

[9] Wu S., Sun J., Li Q., Hood Z.D., Yang S., Su T., Peng R., Wu Z., Sun W., Kent P.R.C. (2020). Effects of surface terminations of 2D Bi_2_WO_6_ on photocatalytic hydrogen evolution from water splitting. ACS Appl. Mater. Interfaces.

[10] Mao W., Shen X., Zhang L., Liu Y., Liu Z., Guan Y.A. (2024). review on Bi_2_WO_6_-based photocatalysts synthesis, modification, and applications in environmental remediation, life medical, and clean energy. Front. Environ. Sci. Eng..

[11] Zhang L., Zhu Y. (2012). A review of controllable synthesis and enhancement of performances of bismuth tungstate visible-light-driven photocatalysts. Catal. Sci. Technol..

[12] Huang H., Zhao J., Du Y., Zhou C., Zhang M., Wang Z., Weng Y., Long J., Hofkens J., Steele J.A. (2020). Direct Z-scheme heterojunction of semicoherent FAP-bBr_3_/Bi_2_WO_6_ interface for photoredox reaction with large driving force. ACS Nano.

[13] Chen T., Liu L., Hu C., Huang H. (2021). Recent advances on Bi_2_WO_6_-based photocatalysts for environmental and energy applications. Chin. J. Catal..

[14] Jiang H., He J., Deng C., Hong X., Liang B. (2022). Advances in Bi_2_WO_6_-based photocatalysts for degradation of organic pollutants. Molecules.

[15] Khedr T.M., Wang K., Kowalski D., El-Sheikh S.M., Abdeldayem H.M., Ohtani B., Kowalska E. (2022). Bi_2_WO_6_-based Z-scheme photocatalysts: Principles, mechanisms and photocatalytic applications. J. Environ. Chem. Eng..

[16] Ren H., Qi F., Labidi A., Allam A.A., Ajarem J.S., Bahnemann D.W., Wang C. (2022). Turning agroforestry waste into value-added fluorescent carbon quantum dots for effective detection of Fe^3+^ in an aqueous environment. ACS EST Engg..

[17] Ren H., Chen Y., Labidi A., Zhao K., Xu X., Othman S.I., Allam A.A., Rudayni H.A., Wang C. (2024). Transforming bio-waste lignin into amine functionalized carbon quantum dots for selective detection of trace Cu^2+^ in aqueous system. Int. J. Biol. Macromol..

[18] Ren H., Labidi A., Sial A., Gao T., Xu X., Wang C. (2024). Carbon quantum dots modified Z and S–Scheme heterojunctions for pharmaceutical contaminants photodegradation: State–of–the–art, benefits, and limitations. Sep. Purif. Technol..

[19] Chen T., Yin D., Zhang X., Zhao F., Khaing K., Deng L., Huang K., Li L., Liu J., Zhang Y. (2019). Fabrication of a novel carbon quantum Dots-Modified 2D heterojunction for highly efficient sunlight photocatalysis. J. Alloys Compd..

[20] He Z., Wang H., Liang M., Ma H., Zhang C., Zhao Y., Qu Y., Miao Z. (2024). Controlled synthesis of spindle-like CoNi_2_S_4_ as electrode material for aqueous energy storage application. Int. J. Hydrogy Energy.

[21] Pirsaheb M., Asadi A., Sillanpää M., Farhadian N. (2018). Application of carbon quantum dots to increase the activity of conventional photocatalysts: A systematic review. J. Mol. Liq..

[22] Kumar P., Dua S., Kaur R., Kumar M., Bhatt G. (2022). A review on advancements in carbon quantum dots and their application in photovoltaics. RSC Adv..

[23] Mei A., Xu Z., Wang X., Liu Y., Chen J., Fan J., Shi Q. (2022). Photocatalytic materials modified with carbon quantum dots for the degradation of organic pollutants under visible light: A review. Environ. Res..

[24] Xiao L., Xu X., Jia Y., Hu G., Hu J., Yuan B., Yu Y., Zou G. (2021). Pyroelectric nanoplates for reduction of CO_2_ to methanol driven by temperature-variation. Nat. Commun..

[25] Kong X.Y., Tan W.L., Ng B.J., Chai S.P., Mohamed A. (2017). Harnessing Vis-NIR broad spectrum for photocatalytic CO_2_ reduction over carbon quantum dots-decorated ultrathin Bi_2_WO_6_ nanosheets. Nano Res..

[26] Yin W., Liu L., Zhang H., Tang S., Chi R. (2020). A facile solvent-free and one-step route to prepare amino-phosphonic acid functionalized hollow mesoporous silica nanospheres for efficient Gd (III) removal. J. Clean. Prod..

[27] Jian L., Wang G., Liu X., Ma H. (2024). Unveiling an S-scheme F-Co_3_O_4_@Bi_2_WO_6_ heterojunction for robust water purification. eScience.

[28] Quan Y., Wang B., Liu G., Li H., Xia J. (2021). Carbonized polymer dots modified ultrathin Bi1_2_O_17_Cl_2_ nanosheets Z-scheme heterojunction for robust CO_2_ photoreduction. Chem. Eng. Sci..

[29] Cheng Y.H., Chen J., Che H.N., Ao Y.H., Liu B. (2022). Ultrafast photocatalytic degradation of nitenpyram by 2D ultrathin Bi_2_WO_6_: Mechanism, pathways and environmental factors. Rare Met..

[30] Ren H., Labidi A., Gao T., Padervand M., Liang X., Wang C. (2024). Efficient conversion of bio-waste lignin into high-value fluorescent nitrogen-modified carbon quantum dots for live-cell imaging. Ind. Crops Prod..

[31] Cai M., Wang C., Liu Y., Yan R., Li S. (2022). Boosted photocatalytic antibiotic degradation performance of Cd_0.5_Zn_0.5_S/carbon dots/Bi_2_WO_6_ S-scheme heterojunction with carbon dots as the electron bridge. Sep. Purif. Technol..

[32] Ren X., Wu K., Qin Z., Zhao X., Yang H. (2019). The construction of type II heterojunction of Bi_2_WO_6_/BiOBr photocatalyst with improved photocatalytic performance. J. Alloys Compd..

[33] Liu X., Yang Y., Li H., Yang Z., Fang Y. (2021). Visible light degradation of tetracycline using oxygen-rich titanium dioxide nanosheets decorated by carbon quantum dots. Chem. Eng. J..

[34] Wu S., Li X., Tian Y., Lin Y., Hu Y. (2021). Excellent photocatalytic degradation of tetracycline over black anatase-TiO_2_ under visible light. Chem. Eng. J..

[35] Zhou L., Dai S., Xu S., She Y., Li Y., Leveneur S., Qin Y. (2021). Piezoelectric effect synergistically enhances the performance of Ti_32_-oxo-cluster/BaTiO_3_/CuS p-n heterojunction photocatalytic degradation of pollutants. Appl. Catal. B Environ..

[36] Liang Y., Xu W., Fang J., Liu Z., Chen D., Pan T., Yu Y., Fang Z. (2021). Highly dispersed bismuth oxide quantum dots/graphite carbon nitride nanosheets heterojunctions for visible light photocatalytic redox degradation of environmental pollutants. Appl. Catal. B Environ..

[37] Dong H., Zhang X., Li J., Zhou P., Yu S., Song N., Liu C., Che G., Li C. (2020). Construction of morphology-controlled nonmetal 2D/3D homojunction towards enhancing photocatalytic activity and mechanism insight. Appl. Catal. B Environ..

[38] Shi Y., Li L., Sun H., Xu Z., Cai Y., Shi W., Guo F., Du X. (2022). Engineering ultrathin oxygen-doped g-C_3_N_4_ nanosheet for boosted photoredox catalytic activity based on a facile thermal gas-shocking exfoliation effect. Sep. Purif. Technol..

[39] Xiaona Jiang S.C., Zhang X., Lanni Qu H.Q., Wang B., Xu B., Huang Z. (2023). Car-bon-doped flower-like Bi_2_WO_6_ decorated carbon nanosphere nanocomposites with. Adv. Compos. Hybrid. Mater..

[40] Zhang M.M., Lai C., Li B.S., Huang D.L., Zeng G.M., Xu P., Qin L., Liu S.Y., Liu X.G., Yi H. (2019). Rational design 2D/2D BiOBr/CDs/g-C_3_N_4_ Z-scheme heterojunction photocatalyst with carbon dots as solid-state electron mediators for enhanced visible and NIR photocatalytic activity: Kinetics, intermediates, and mechanism insight. J. Catal..

[41] Zhou Q., Zhang L., Zhang L., Jiang B., Sun Y. (2022). In-situ constructed 2D/2D ZnIn_2_S_4_/Bi_4_Ti_3_O_12_ S-scheme heterojunction for degradation of tetracycline: Performance and mechanism insights. J. Hazard. Mater..

[42] Ye L., Sun S., Yang X., Chen X., Yang B., Yun D., Yu X., Yang M., Yang Q., Liang S. (2023). Mechanism insight into the enhanced photocatalytic purification of antibiotic through encapsulated architectures coupling of crystalline Cu_2_O/amorphous TiFe layer double hydroxide. J. Mater. Sci. Technol..

[43] Ren H., Qi F., Labidi A., Zhao J., Wang H., Xin Y., Luo J., Wang C. (2023). Chemically bonded carbon quantum dots/Bi_2_WO_6_ S-scheme heterojunction for boosted photocatalytic antibiotic degradation: Interfacial engineering and mechanism insight. Appl. Catal. B Environ..

[44] Tada H., Mitsui T., Kiyonaga T., Akita T., Tanaka K. (2006). All-solid-state Z-scheme in CdS-Au-TiO_2_ three-component nanojunction system. Nat. Mater..

[45] Wan J., Xue P., Wang R., Liu L., Liu E., Bai X., Fan J., Hu X. (2019). Synergistic effects in simultaneous photocatalytic removal of Cr (VI) and tetracycline hydrochloride by Z-scheme Co_3_O_4_/Ag/Bi_2_WO_6_ heterojunction. Appl. Surf. Sci..

[46] Wen T.Y., Wei G.Y., Ying K.X., Ng B.J., Yong S.T., Chai S.P. (2019). Fabrication of Bi_2_WO_6_/Cu/WO_3_ all-solid-state Z-scheme composite photocatalyst to improve CO_2_ photoreduction under visible light irradiation. ChemCatChem.

[47] Dang V., Jr J., Annadurai T., Bui T., Tran H., Lin L., Doong R. (2021). Indirect Z-scheme nitrogen-doped carbon dot decorated Bi_2_MoO_6_/g-C_3_N_4_ photocatalyst for enhanced visible-light-driven degradation of ciprofloxacin. Chem. Eng. J..

[48] Zhou Y., Zhang Y., Lin M., Long J., Zhang Z., Lin H., Wu J., Wang X. (2015). Monolayered Bi_2_WO_6_ nanosheets mimicking heterojunction interface with open surfaces for photocatalysis. Nat. Commun..

[49] Zhang Z., Yates T. (2012). Band bending in semiconductors: Chemical and physical consequences at surfaces and interfaces. Chem. Rev..

[50] Chen Q., Ning S., Yang J., Wang L., Yin X., Wang X., Wei Y., Zeng D. (2024). In situ interfacial engineering of CeO_2_/Bi_2_WO_6_ heterojunction with improved photodegradation of tetracycline and organic dyes: Mechanism insight and toxicity assessment. Small.

